# Cardiovascular and psychological responses to voluntary recall of trauma in posttraumatic stress disorder

**DOI:** 10.1080/20008198.2018.1472988

**Published:** 2018-06-04

**Authors:** Chia-Ying Chou, Roberto La Marca, Andrew Steptoe, Chris R. Brewin

**Affiliations:** a Department of Clinical, Educational & Health Psychology, University College London, London, UK; b Department of Psychology, Universität Zürich, Zürich, Switzerland; c Department of Behavioural Science and Health, University College London, London, UK

**Keywords:** Heart rate variability, heart rate, PTSD, memory recall, flashbacks, depersonalisation/derealisation, 心率变异性, 心率, 创伤后应激障碍, 回忆, 闪回, 人格解体/现实解体, Variabilidad de la frecuencia cardiaca, frecuencia cardiaca, TEPT, evocación, flashbacks, despersonalización/desrealización, • Initial heart rate increase during the recall of trauma, that declines over time., • Parasympathetic activation indicates state depersonalization/derealization., • Heart rate elevation indicates fear and flashbacks.

## Abstract

Voluntary recall of trauma is a key element in exposure-based psychotherapies and can trigger spontaneous dissociative responses such as flashbacks, depersonalisation, and derealisation. In order to examine the associations between cardiovascular and psychological responses to voluntary recollection of trauma, individuals with PTSD recalled a traumatic memory. Heart rate and heart rate variability were recorded continuously and the episodes when different forms of dissociation were experienced during the recall were identified. A significant increase in parasympathetic activity was found during trauma recall, with greater parasympathetic dominance being indicative of greater state depersonalisation/derealisation. Whereas overall decreases in heart rate during trauma recall were associated with increased fear and perceived threat, flashbacks were accompanied by short-term increases in heart rate. These findings demonstrate different types of cardiovascular responses associated with different psychological experiences during trauma recall. Future research directions were discussed.

## Abstract (es)

El recuerdo voluntario del trauma es un elemento clave en las psicoterapias basadas en la exposición y puede gatillar respuestas disociativas espontáneas como flashbacks, despersonalización y desrealización. Con el fin de examinar las asociaciones entre las respuestas cardiovascular y psicológica al recuerdo voluntario del trauma, individuos con TEPT evocaron una memoria traumática. Se midió en forma continua su frecuencia cardiaca y la variabilidad de la misma y se identificó los episodios en que experimentaron diferentes formas de disociación durante la evocación. Se encontró un incremento significativo en la actividad parasimpática durante el recuerdo del trauma, siendo una mayor dominancia parasimpática indicadora de un mayor estado de despersonalización/desrealización. Mientras que una disminución general en la frecuencia cardiaca durante el recuerdo del trauma se asoció a un incremento del miedo y percepción de amenaza, los flashbacks estaban acompañados de incrementos breves de la frecuencia cardiaca. Estos hallazgos demuestran que diferentes tipos de respuesta cardiovascular se asocian a diferentes experiencias psicológicas durante el recuerdo del trauma. Se discutieron futuras direcciones para la investigación.

## Abstract (zh)

自主回忆创伤是以暴露为基础的心理治疗中的一个关键因素，并且可能引起自发的分离反应（如闪回、人格解体和现实解体）。为了检查自发性创伤回忆過程中的心血管和心理反應之間的关系，本研究請PTSD患者回忆一段创伤记忆，在回憶過程中持续记录心率和心率变异性，并发生的不同形式的解离。本研究發現创伤回忆期间副交感神经活性显着增加，副交感神经活性越強勢人格解体/现实解体状态越严重。创伤回忆期间的整體心率下降与恐惧感和威胁感呈現正相关，而閃回則與心率上升呈顯著相關。这些研究结果呈現了与创伤回忆期间不同的心理体验相关的不同心血管反應。文中讨论了未来的研究方向。且辨別和記錄此過程中此外，

Voluntary recall of the traumatic memory is the main technique of exposure-based psychotherapy for posttraumatic stress disorder (PTSD). During voluntary recall, full activation of vivid images and raw emotions, as well as the ability to hold these materials in focal attention and integrate them into a meaningful cognitive structure are key elements in successful therapy (Brewin, Gregory, Lipton, & Burgess, 2010; Foa, Steketee, & Rothbaum, 1989). Accordingly, it is important to investigate ways of detecting emotional arousal (e.g. fear and perceived threat) and different states of consciousness such as depersonalisation, derealisation, and flashbacks (Lanius, 2015) during voluntary recall of trauma among individuals with PTSD.

## 0.1. Heart rate as an index of emotional arousal

Psychological and physiological states in response to trauma reminders or cued recollection of trauma (e.g. script-driven imagery: Lang, Levin, Miller, & Kozak, 1983) have been widely studied (e.g. Hopper, Frewen, Sack, Lanius, & Van Der Kolk, 2007; Pitman, Orr, Forgue, De Jong, & Claiborn, 1987). When individuals with PTSD underwent cued recall of trauma, or were exposed to reminders that triggered involuntary traumatic memories, increased heart rate (HR), a sign of emotional arousal (Blechert, Michael, Grossman, Lajtman, & Wilhelm, 2007), has consistently been found (e.g. Arditi-Babchuk, Feldman, & Gilboa-Schechtman, 2009; O’Kearney & Parry, 2014; Pitman et al., 1990).

Few studies (e.g. Cohen et al., 2000, 1998; Halligan, Michael, Wilhelm, Clark, & Ehlers, 2006; Keary, Hughes, & Palmieri, 2009) have examined cardiovascular responses during voluntary recall of traumatic memories (i.e. recall without the presentation of reminders). A lower HR reactivity to the recollection of traumatic memories among individuals with PTSD, compared to that of healthy controls or other clinical populations, has been found in most of these studies (Cohen et al., 2000, 1998; Halligan et al., 2006). The study by Halligan and colleagues (Halligan et al., 2006), for example, has found that trauma victims with PTSD showed smaller HR increases (relative to baseline) compared to those without PTSD while voluntarily recalling traumatic memories. Moreover, smaller HR increases during trauma recall were found to predict poorer recovery in PTSD symptoms six months later. These findings of lowered cardiovascular reactivity are reminiscent of the proposal that limited emotional arousal impedes the processing of traumatic memories (Foa, Molnar, & Cashman, 1995).

## 0.2. Heart rate as an index of different states of consciousness

In addition to emotional arousal, different states of consciousness affect the degree of traumatic memory processing. Among them, two dissociative states, depersonalisation and derealisation, have been recognised as distinct features of a subtype of PTSD in DSM-5 (American Psychiatric Association, 2013). They were also identified as predictors of poorer responses to exposure therapies due to their inhibiting effects on conscious engagement during traumatic memory processing (Lanius, Brand, Vermetten, Frewen, & Spiegel, 2012; Lanius et al., 2010).

Studies have consistently associated depersonalisation and derealisation with lower levels of physiological arousal. In one of the first studies to demonstrate this, Griffin, Resick, and Mechanic (1997) reported that rape victims with greater peri-traumatic depersonalisation/derealisation showed lower HR when verbally recalling their trauma. Similar associations were found in another study that recruited adolescents with childhood abuse experiences (Koopman et al., 2004). Concurrent depersonalisation/derealisation and HR during involuntary retrieval of trauma memories (triggered by trauma scripts) were examined in a later study (Sack, Cillien, & Hopper, 2012). It was found that individuals experiencing both flashbacks and high depersonalisation/derealisation during the procedure exhibited lower HR, compared to those experiencing flashbacks but low depersonalisation/derealisation. These studies demonstrated the association between depersonalisation/derealisation and decreased HR reactivity during traumatic memory processing.

No study to our knowledge has assessed depersonalisation/derealisation and concurrent cardiovascular reactivity during voluntary recall of traumatic memories. Nor is much known about flashbacks, and more specifically their cardiovascular profile, despite them being a cardinal symptom of PTSD in ICD-11 (Brewin, 2015; Karatzias et al., 2017). Like depersonalisation/derealisation, flashbacks are common symptoms experienced by individuals with PTSD. They have been suggested to be a product of an imbalance of encoding into contextualised and image-based memory systems, with a different neural basis from regular autobiographical memories (Brewin et al., 2010). Examination of potential cardiovascular indices of flashbacks as well as of depersonalisation/derealisation during voluntary recall of trauma is likely to inform knowledge of the processes underlying exposure therapy.

## 0.3. Heart rate variability and traumatic memory processing

Flexibility of the autonomic nervous system, indicated by alterations in both the sympathetic nervous systems (SNS) and parasympathetic nervous system (PNS) in response to stress, has been associated with better psychological adjustment (Appelhans & Luecken, 2006; Berntson, Norman, Hawkley, & Cacioppo, 2008; Thayer, Ahs, Fredrikson, Sollers, & Wager, 2012). While heightened SNS activation has been suggested to underlie the fight or flight response (Thayer & Lane, 2009), elevated PNS activation has been associated with increased concentration or a state freezing response under extreme stress (Bradley & Lang, 2007; Chou, La Marca, Steptoe, & Brewin, 2014; Hansen, Johnsen, & Thayer, 2003).

Heart rate variability (HRV) has gained research attention due to its ability to provide separate estimates of PNS and SNS activity. Applying frequency domain analysis (Cohen, Matar, Kaplan, & Kotler, 1999), the high frequency component of HRV (HF-HRV) is considered as a marker of PNS activity. The low frequency component (LF-HRV) is suggested by some as a marker of SNS activity, and by others as being influenced both by the PNS and SNS. Consequently, some believe the LF/HF ratio reflects SNS/PNS balance, and others believe it indexes SNS activity (Task Force of the European Society of Cardiology and the North American Society of Pacing and Electrophysiology, 1996).

Among studies assessing HRV during voluntary recall of trauma, when reliving was not specifically required significant changes in HRV (relative to a resting baseline) were not found (Cohen et al., 2000, 1998). In contrast, when vivid recall of details was asked for, a significant decrease in HF-HRV was reported, and the reduction was greater among individuals with PTSD than healthy controls (Keary et al., 2009). These studies tentatively suggest an association between varying levels of HRV reactivity and different level of emotional arousal and states of consciousness during voluntary recall of trauma.

## 0.4. The current study

The goal of the current study was to examine the relationships between psychological and cardiovascular responses during voluntary recall of trauma – a key component of exposure-based therapies. To this end, the current study assessed HR, HRV, and emotional arousal (i.e. levels of fear and perceived threat) during a voluntary traumatic memory recall protocol mimicking the methods used in exposure-based therapies. HR, HRV, and emotional arousal were also assessed during the recall of a neutral memory, which was chosen as the control condition in order to account for the cardiovascular variation produced by speaking. We hypothesised an increase in HR during trauma recall (compared with HR during neutral recall), and that the level of HR increase would be positively correlated with levels of emotional arousal (i.e. fear and threat), since increase in HR has been associated with strong emotional arousal and fight-or-flight responses (e.g. Blechert et al., 2007; Brisinda et al., 2014). In contrast, we expected levels of depersonalisation/derealisation to be associated with decreased HR based on the previous literature (e.g. Sack et al., 2012). As for the HRV measures, we expected a decrease in HF-HRV (PNS activation), and that the level of decrease to be associated with greater emotional arousal (i.e. greater fear and perceived threat) based on previous work (Keary et al., 2009).

Additionally, to explore the relationship between psychological and cardiovascular responses during trauma recall at a moment-to-moment level, we adopted a method used and validated in previous studies (Brewin, Huntley, & Whalley, 2012; Hellawell & Brewin, 2002, 2004; Whalley et al., 2013). This method involves asking participants to review the video recorded during their own trauma recall process, and to identify specific episodes during the recall when different states of consciousness were experienced. The types of states we studied included: depersonalisation/derealisation; flashbacks; and a mixture of both (referred to as a ‘mixed period’ hereafter). The remaining recall period corresponded to a non-dissociative state, i.e. an ordinary recall episode without any of the above altered states of consciousness (Lanius, 2015). We hypothesised that higher HR would be associated with flashbacks and mixed periods of trauma recall since these states may involve greater emotional arousal.

## 1. Methods

### 1.1. Participants

The participants were recruited through referrals from the Traumatic Stress Clinic and the Improving Access to Psychological Therapies Services of the Camden and Islington NHS Foundation Trust in London, as well as through advertisement to the general public. After volunteers gave permission to the experimenter to contact them, a detailed explanation regarding the study was given, and the inclusion and exclusion criteria were checked over the phone. The inclusion criteria included a current diagnosis of PTSD based on the Structured Clinical Interview for DSM-IV (SCID), age between 20 and 65 years, and fluent in the English language. The exclusion criteria consisted of a current diagnosis of psychotic or substance-related disorder based on the SCID, a current high suicidal risk, or a current diagnosis of cardiovascular or neurological disease.

**Figure 1.**
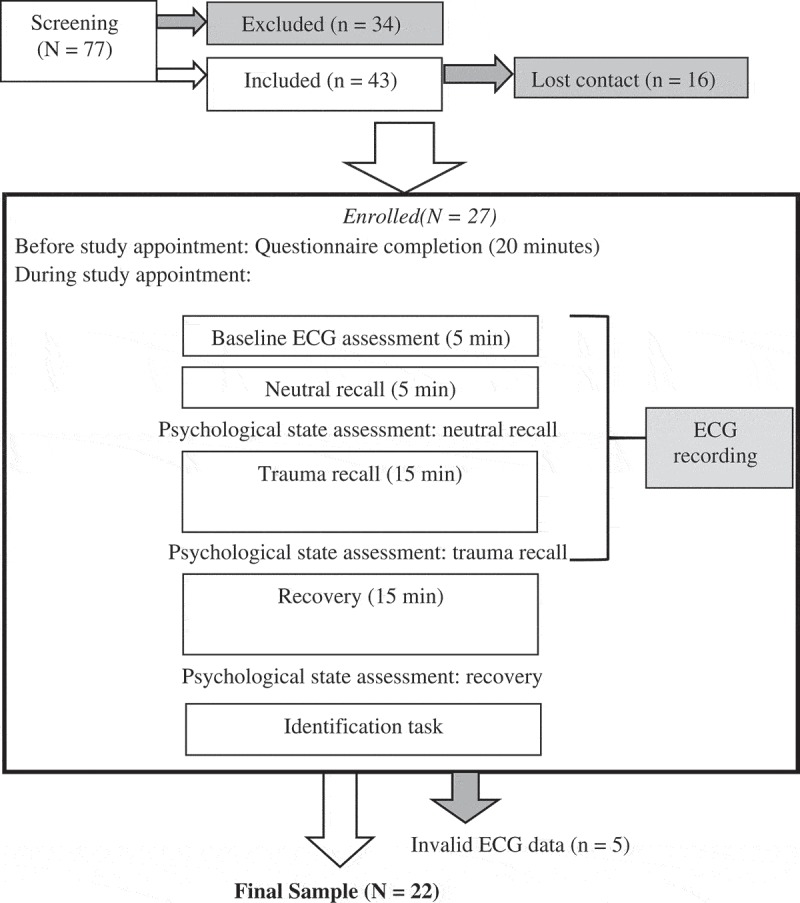
Procedures.

As shown in Figure 1, 77 volunteers went through the screening procedures. Thirty-one of them did not meet the diagnostic criteria for PTSD; one met a current diagnosis of schizophrenia; one met a current diagnosis of cannabis dependence; and one had a brain injury and suffered from epilepsy. Among the 43 eligible volunteers, 16 lost contact before the study appointment. All of the 27 participants who came to the appointment completed and were paid £10 per hour for participation. Data from five were excluded at the analysis stage due to a high number of artefacts in the ECG data (i.e. more than 3% corrected R-R intervals; Hodson, Harnden, Roberts, Dennis, & Frayn, 2010). This resulted in a final sample of 22 (7 males), aged between 25 and 61 years. Most participants experienced multiple types of trauma (*M* = 4.45, *SD* = 2.28). As shown in Table 1, one participant missed the questionnaires assessing body mass index, years in education, duration of therapy, and the psychological state measures after neutral recall, hence N of these measures are 21. This participant and another participant missed the PTSD symptom severity assessment, hence N of this measure is 20.

**Table 1.** Summary of study variables.

	N	Mean (SD)
**Background information**		
Age	22	42.36 (10.31)
Body Mass Index (kg/m^2^)	21	24.91 (6.32)
Years in education	21	15.00 (2.35)
Duration of therapy^a^	21	2.00 (1.18)
PTSD symptom severity (0–51)	20	32.60 (10.19)
**Psychological state responses**		
**Fear (0–10)**		
Neutral recall	21	1.90 (2.70)
Trauma recall	22	4.18 (3.45)
Recovery	22	2.27 (2.14)
**Threat (0–10)**		
Neutral recall	21	1.52 (2.58)
Trauma recall	22	3.00 (3.60)
Recovery	22	2.09 (2.64)
**State-like depersonalisation/derealisation (0–76)**		
Neutral recall	21	15.38 (18.63)
Trauma recall	22	22.59 (19.67)
Recovery	22	14.68 (17.04)
**Cardiovascular responses**		
**Heart rate (beats per minute)**		
Neutral recall	22	76.96 (7.72)
Trauma recall	22	77.27 (7.97)
**Low frequency heart rate variability (ms^2^)**		
Neutral recall	22	1315.56 (1572.72)
Trauma recall	22	1391.51 (1587.15)
**High frequency heart rate variability (ms^2^)**		
Neutral recall	22	243.72 (320.55)
Trauma recall	22	534.43 (488.05)
**Low frequency/high frequency ratio**		
Neutral recall	22	6.66 (5.04)
Trauma recall	22	6.45 (4.29)

^a^ 1 = never, 2 = 1–10 weeks, 3 = 11 weeks-1 year, 4 = more than 1 year.

Note: Ns vary due to missing data.

### 1.2. Memory recall

The voluntary memory recall task was developed with reference to a previous method (Halligan et al., 2006). Participants were first asked to recall and speak about a neutral memory for five minutes. This served as a control condition and a preparation for the recollection of trauma. After the neutral retrieval, participants were asked to recall and speak about a piece of traumatic memory for 15 minutes. This longer period was based on a previous study of trauma narratives by Hellawell and Brewin (2002), but it was not thought to be realistic to recall a neutral memory for a comparable time. The instructions for both recalls were as below. ECG data and videotape recordings were collected throughout both recalls.

(1)*Instructions for neutral recall*

*There are many routines in daily life, which are familiar to us, but do not cause significant emotional reactions to us. Examples of routines include tidying the house, doing the laundry, walking or taking a bus/tube/train ride to work or supermarkets. Please choose a routine that you are familiar with, but do not emotionally react to. When you are ready, with your eyes open, please take yourself back to the time when you last did it. Begin just before it started. Go through everything that happened from start to finish. Include details about the surroundings. Describe everything you remember seeing, smelling, hearing, doing, feeling, and thinking about at each point in time.*

(2)*Instructions for trauma recall*

*In the questionnaire you completed, you chose a traumatic event which distresses you the most. When you are ready, with your eyes open, I would like you to take yourself back to the time of the event and remember it as vividly as you can. Begin just before it started. Go through everything that happened from start to finish. Include details about the surroundings. Describe everything you remember seeing, smelling, hearing, doing, feeling, and thinking about at each point in time.*

## 2. Identification of depersonalisation/derealisation and flashbacks during trauma recall

Participants were asked to watch the video (or listen to the audio) taken during the trauma recall, in order to identify the exact periods when depersonalisation/derealisation, flashbacks, or a mixture of both were experienced. The instructions are as below.

*Please identify the times when these two mental states occurred to you while you were recalling the memory of the traumatic incident.*

(1)*Depersonalisation/derealisation*

*During the recall, were there times when you felt as if the surrounding environment (this room) was unreal, or when you felt as if you were looking at things from outside of your body, or when you felt blanked out and it was difficult to make sense of what was going on?*

(2)*Flashbacks*

*During the recall, were there times when you felt that the original vivid feelings or memories of the event (e.g., images, sounds, smells, emotional and physiological feelings) were coming back to you, as if you were experiencing the event again?*

### 2.1. Cardiovascular data acquisition

The ECG signal was recorded from two disposable electrodes attached to participants’ chests (one adhered at the point where the sternum meets the space between ribs four and five; the other adhered at the point where the anterior axillary line meets the space between ribs five and six). It was sampled continuously at 512 Hz with the Actiwave Cardio system (Camntech, Cambridge, UK). The ECG data were examined and derived using VivoSense software (VivoNoetics, San Diego, CA, USA). Artefacts in the data related to misdetected heart beats were easily recognised, since they stood out from the average HR curve. These artefacts were manually detected and interpolated using the facility provided by the software (Halligan et al., 2006). Data with more than 3% corrected R-R intervals were excluded (Hodson et al., 2010).

HR and HRV parameters were derived for the resting baseline (five minutes), and the neutral and trauma recall periods. The frequency domain indices of HRV were selected. The power spectrum density of the R-R intervals was computed using a Fast Fourier Transformation, which decomposes the variance in the frequency domain (ms^2^/Hz). Spectral power was divided into LF-HRV (0.04–0.14 Hz), HF-HRV (0.15–0.40 Hz), and LF/HF-ratio was calculated following the guidelines for frequency-domain computations of HRV (Task Force of the European Society of Cardiology and the North American Society of Pacing and Electrophysiology, 1996).

### 2.2. Psychological measures

#### 2.2.1. Structured clinical interview for DSM-IV (SCID)

The SCID (First, Gibbon, Spitzer, Williams, & Benjamin, 1997) is a standardised semi-structured interview designed to identify the presence of Axis I psychopathology.

#### 2.2.2. Posttraumatic stress diagnostic scale (PDS)

The PDS (Foa, 1995) is a 49-item self-report measure of traumatic experiences and related PTSD symptoms. The first sections asked respondents to identify potential traumatic events, indicate which of these currently troubles them the most, and rate their subjective reactions at the time. In the third and fourth sections, Criteria B, C, D, and E for PTSD are rated. PTSD symptom severity is indicated by the sum of all the items for Criteria B, C, and D. The scores range between 0 and 51, with higher scores indicating greater severity.

#### 2.2.3. Dissociative state scale (DSS)

The DSS is a 19-item self-report measure assessing state depersonalisation/derealisation adapted from the Clinician Administered Dissociative State Scale (CADSS; Bremner et al., 1998). Participants are required to rate on a 0-to-4 scale (not at all to extremely) their feelings at a particular moment in time. The total scores range between 0 and 76, with higher scores indicating greater dissociation. Its validity has been supported (Bremner et al., 1998).

#### 2.2.4. Mood rating scale

The mood rating scale was designed for the current study to assess the states of fear and perceived threat. Participants were asked to rate their levels of fear and perceived threat on an 11-point visual analogue scale (0 = not at all, 10 = extremely).

### 2.3. Procedures

The procedure used in the current study is shown in Figure 1. Before the study appointment, participants gave verbal informed consent, and completed the PDS and a short survey on the use of non-prescribed substances. Participants were asked to avoid illicit drugs and alcohol for seven days, vigorous exercise for three days, and caffeine and nicotine for three  hours before the appointment. Medication was advised to be taken as usual. At the beginning of the study appointment, written informed consent was obtained. Two participants who were unwilling to be videotaped agreed to be audiotaped instead. After the ECG and video recording set-up, resting baseline ECG was assessed, followed by the neutral and trauma memory recall. Psychological states were assessed immediately after both recall periods, and at the end of a 15-minute recovery period. Next, the identification of depersonalisation/derealisation and flashback episodes was performed. The current study was approved by the London Bridge National Research Ethics Service Committee.

### 2.4. Analytic strategy

All statistical analyses were performed with SPSS version 21 (SPSS Inc, Chicago, IL, USA). Outliers were handled using recommended methods (Tabachnick & Fidell, 1996) employed in a previous study (Chou et al., 2014), according to which scores greater than three standard deviations above the mean were changed to one unit larger than the greatest non-extreme score of the given variable, whereas scores smaller than three standard deviations below the mean were changed to one unit smaller than the smallest non-extreme score. For example, an outlying score of 27.0 would be changed to 11.5 if the highest non-extreme score was 10.5. This procedure was applied to one data point on perceived threat during neutral recall, one data point on HF-HRV during neutral, and two data points on HF-HRV during trauma recall. Normality of distribution was examined by dividing the absolute values of skewness by the standard error of skewness. For variables with values larger than three from this calculation, square root transformation was performed.

One-way repeated-measures ANOVAs were performed to examine the variance of psychological states (i.e. fear, threat, state depersonalisation/derealisation) across different phases of the study (i.e. neutral recall, trauma recall, and recovery period). The percentages of time when flashbacks, depersonalisation/derealisation, and mixed periods occurred during trauma recall were calculated by dividing the total duration of the given condition by the total duration of trauma recall (i.e. 15 minutes).

Paired sample t-tests were applied to compare cardiovascular activity during the trauma recall with that during neutral recall. For this part of the analysis, mean HR during trauma and neutral recall was derived directly by the VivoSense software (VivoNoetics, San Diego, CA, USA). Since the lengths of the two recall periods (i.e. 5 minutes vs. 15 minutes) were different, we separated the 15-minute trauma recall into three successive five-minute segments. Mean HRV during trauma recall was then calculated by averaging the mean HRV of the three five-minute segments in the recall. This method was applied to avoid biased comparison on variability between data embedded in different lengths of measuring periods (Task Force of the European Society of Cardiology and the North American Society of Pacing and Electrophysiology, 1996). Next, Pearson’s correlations were applied to examine the associations between variations in cardiovascular activities and variations in psychological states brought about by trauma recall (i.e. difference scores calculated by deducting levels during the neutral recall from levels during the trauma recall).

#### 2.4.1. Exploratory analyses

To explore the potential effects of time and of the difference in recall durations, repeated-measures ANOVAs were conducted to compare HR and all HRV measures across baseline, neutral recall, and three segments (i.e. first, second, and third five minutes) of the trauma recall. Further, to explore HR fluctuation associated with flashbacks, depersonalisation/derealisation, and mixed periods, compared with non-dissociative periods, paired sample t-tests were conducted. In these comparisons, mean HR during the first five seconds of the targeted episodes was used and compared with mean HR during the last five seconds of the non-dissociative periods preceding the targeted episodes. For example, only mean HR during the first five seconds when one started to have a flashback was calculated and compared with the five seconds of non-dissociative recall right before the flashback happened. This method was used in order to make allowance for the general decline in HR, which was associated with the overall heightened activation of the PNS, over the entire course of the trauma recall (see the Results section for details).

## 3. Results

### 3.1. Sample characteristics

Among our participants, the elapsed time since the most severe trauma was between three and five years on average. The mean score on the PDS (see Table 1) fell in the range of moderate to severe PTSD symptoms (21–35; Foa, 1995). Comorbidity in the sample included major depressive disorder (*n* = 10), generalised anxiety disorder (*n* = 5), obsessive compulsive disorder (*n* = 3), specific phobia (*n* = 3), social phobia (*n* = 2), agoraphobia (*n* = 2), and panic disorder with (*n* = 1), and without agoraphobia (*n* = 1). Medications taken within 24 hours prior to the study appointment included Amitriptyline, Clonazepam, Diazepam, Levothyroxine, Methadone, Methazolamide, Panadol, Paracetamol, Phenergan, Thyroxine, Ventolin, VESIcare, Zopiclone (*n* = 1 for the abovementioned), as well as Propranolol, Paroxetine, Baclofen, Citalopram, Ibuprofen (*n* = 2 for the abovementioned), and Codeine (*n* = 3).

### 3.2. Psychological states

Descriptive data for the psychological state measures at different phases are summarised in Table 1. Significant quadratic effects of time were found for state depersonalisation/derealisation (*F*(1, 20) = 35.96, *p *< .001, η_p_^2^ = .64), fear (*F*(1, 20) = 12.54, *p *< .01, η_p_^2^ = .39), and threat (*F*(1, 20) = 6.13, *p *< .05, η_p_^2^ = .24). Post hoc analyses showed that state depersonalisation/derealisation was significantly higher after trauma recall than neutral recall and the recovery period (both *ps* < .001). Fear was significantly stronger after trauma recall than neutral recall (*p *< .001), and the recovery period (*p *< .01). Similarly, threat was significantly stronger after trauma recall than neutral recall (*p *< .01), and the recovery period (*p *< .05). Moreover, threat was still stronger after the recovery period than after neutral recall (*p *< .05).

### 3.3. Flashbacks and depersonalisation/derealisation during trauma recall

The traumatic incidents recalled included sexual assaults (*n* = 5), physical assaults (*n *= 3), kidnapping and imprisonment (*n* = 3), tragic or sudden death of a loved one (*n* = 3), car accident (*n* = 2), childhood sexual abuse (*n* = 2), childhood emotional abuse and neglect (*n* = 2), childhood physical abuse (*n* = 1), and war (*n* = 1).

Most participants (*n* = 20) reported flashbacks. Among them, the mean number of periods of flashback was 7.2 (Minimum = 1, Maximum = 18, *SD* = 5.28), with the shortest sequence lasting 5 seconds, and the longest sequence lasting 11 minutes and 34 seconds (*M *= 45.68, *SD *= 86.90 seconds). The mean percentage of time during trauma recall when flashbacks were experienced was 38.12% (*SD* = 28.93%). Most flashback sequences occurred after a period of non-dissociative recall (83.10%). Some of them followed mixed periods (12.68%), and very few of them followed depersonalisation/derealisation (4.23%).

Nine participants reported depersonalisation/derealisation during trauma recall. Among them, the mean number of periods of depersonalisation/derealisation was five (Minimum = 1, Maximum = 22, *SD* = 6.67), with the shortest sequence lasting three seconds, and the longest sequence lasting six minutes (*M *= 23.76, *SD *= 53.07 seconds). The mean percentage of time within the trauma recall when depersonalisation/derealisation occurred was 13.75% (*SD* = 11.69%). Most depersonalisation/derealisation occurred after either a period of non-dissociative recall (44.44%), or a mixed period (42.22%), and only rarely followed flashbacks (13.33%).

Ten participants reported mixed periods during trauma recall (seven of them also reported depersonalisation/derealisation). Among the 10 participants, the mean number of mixed periods was 5.67 (Minimum = 1, Maximum = 19, *SD* = 5.79), with the shortest sequence lasting two seconds, and the longest sequence lasting 78 seconds (*M *= 15.59, *SD *= 13.94 seconds). The mean percentage of time within the trauma recall period when mixed periods occurred was 8.76% (*SD* = 7.60%). Similar proportions of mixed periods occurred after a period of non-dissociative recall (37.74%), flashbacks (32.08%), and depersonalisation/derealisation (30.19%).

### 3.4. HR, HRV, and psychological states in response to trauma recall

Comparing cardiovascular responses between trauma recall and neutral recall, our data showed a significantly higher HF-HRV level during trauma recall (*t*(21) = 2.18, *p* < .05, Cohen’s *d* = .62). In contrast, differences in HR (*t*(21) = .17, *p* = .86, Cohen’s *d* = .04), LF-HRV (*t*(21) = 1.20, *p* = .24, Cohen’s *d* = .26), and LF/HF-ratio (*t*(21) = .37, *p* = .72, Cohen’s *d* = .08) were nonsignificant (descriptive data summarised in Table 1). In terms of the associations with the state psychological measures, as shown in Table 2, a greater reduction in HR during trauma recall (relative to neutral recall) was significantly correlated with greater increases in fear and threat. Moreover, a greater decrease in LF/HF-ratio during trauma recall was significantly correlated with a greater increase in state depersonalisation/derealisation.

**Table 2.** Correlations between changes in psychological and cardiovascular measures (all *df* = 21).

	∆State dissociation	∆Fear	∆Threat
∆HR	-.23	-.53*	-.44*
∆HF-HRV	.16	.18	.16
∆LF-HRV	-.28	-.03	.03
∆LF/HF-ratio difference	-.53*	-.12	-.26

* *p* < .05.

Note: ∆ refers to changes scores, which were calculated by deducting the mean value during neutral recall from that during trauma recall; HR = heart rate; HF-HRV = high frequency heart rate variability; LF-HRV = low frequency heart rate variability; LF/HF-ratio = low frequency/high frequency heart rate variability ratio.

#### 3.4.1. Exploratory analyses and findings

**Figure 2.**
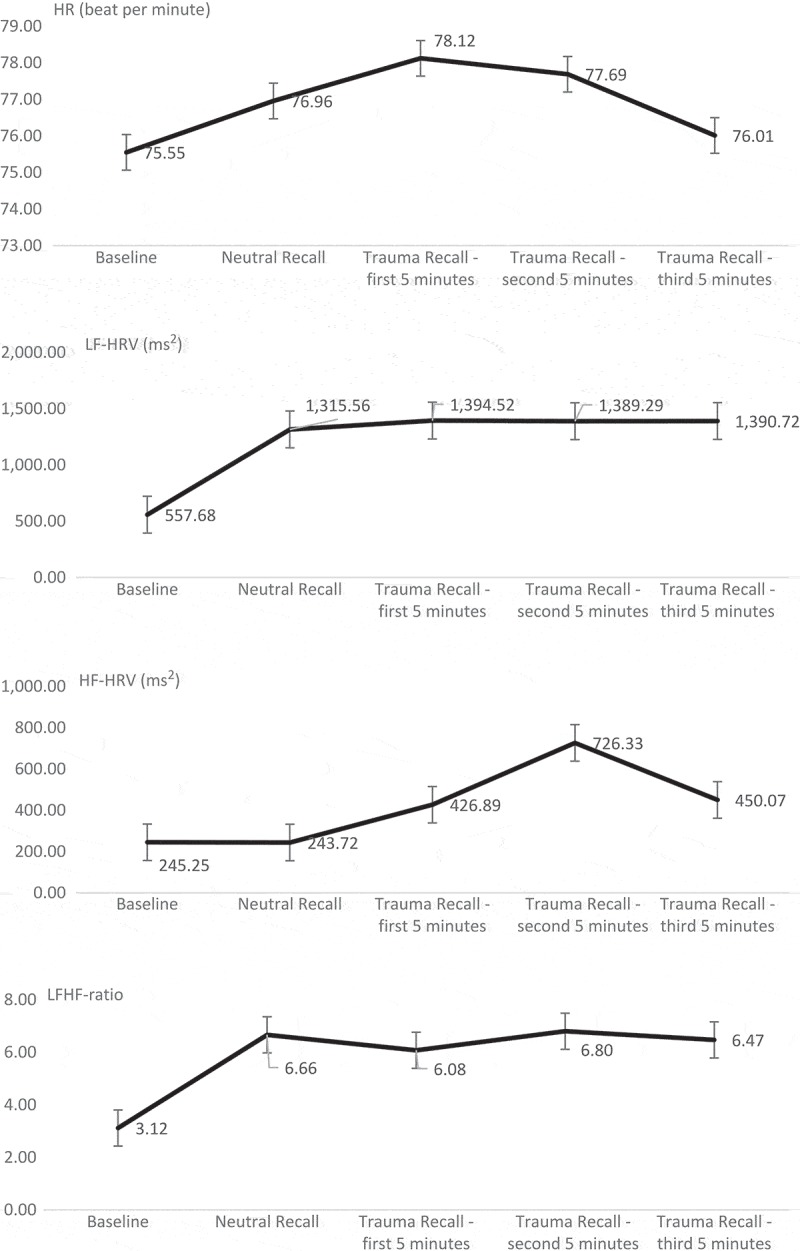
Means and standard errors of cardiovascular measures across study phases.

Bearing in mind the potential effects of time and of the difference in duration of the two recall tasks, variations in different cardiovascular measures across the study phases (i.e. baseline, neutral recall, three five-minute segments of trauma recall) were examined (see Figure 2). Repeated-measures ANOVAs showed a significant effect of time on HR (*F*(4, 76) = 3.10, *p* < .05, η_p_^2^ = .14). Post hoc analyses found that HR during the first five minutes of trauma recall was significantly higher than baseline (*p* < .05). Moreover, HR during the last five minutes of trauma recall was significantly lower than HR during the first and second five minutes (both *p* < .01). Significant effects of time on LF-HRV (*F*(4, 76) = 5.84, *p* < .05, η_p_^2^ = .24) and LF/HF-ratio (*F*(4, 76) = 7.94, *p* < .001, η_p_^2^ = .30) were also found, but not on HF-HRV (*F*(4, 76) = 1.18, *p* = .29, η_p_^2^ = .06). Post hoc analyses indicated that LF-HRV significantly increased during neutral recall from baseline (*p* < .05). LF-HRV of all the five-minute trauma recall segments were significantly higher than baseline as well (*p* < .05 for the first segment, *p* < .01 for the second and third segments). Similarly, LF/HF-ratio significantly increased after neutral recall began (*p* < .001 for all phases compared with baseline). However, differences in LF-HRV and LF/HF-ratio between the trauma recall segments and neutral recall were not significant.

The second-by-second fluctuation of mean HR in response to flashbacks was also examined. Mean HR during the first five seconds of flashback periods (i.e. seconds 6–10; *M* = 76.52, *SD* = 8.68) was significantly higher than mean HR during the five seconds of non-dissociative recall periods just prior to the flashback occurring (i.e. seconds 1–5; *M* = 74.92, *SD* = 7.72; *t*(19) = 2.37, *p* < .05, Cohen’s *d* = .56). The length (i.e. five seconds) was determined considering varying lengths of flashbacks, and the maximum data available, since > 98% of the flashbacks lasted at least five seconds. Among the few participants that experienced depersonalisation/derealisation and/or mixed episodes, only five experienced the former and three experienced the latter with non-dissociative recall periods just prior to these states. Therefore, too few data points were available to carry out the same analysis for depersonalisation/derealisation and mixed episodes.

**Figure 3.**
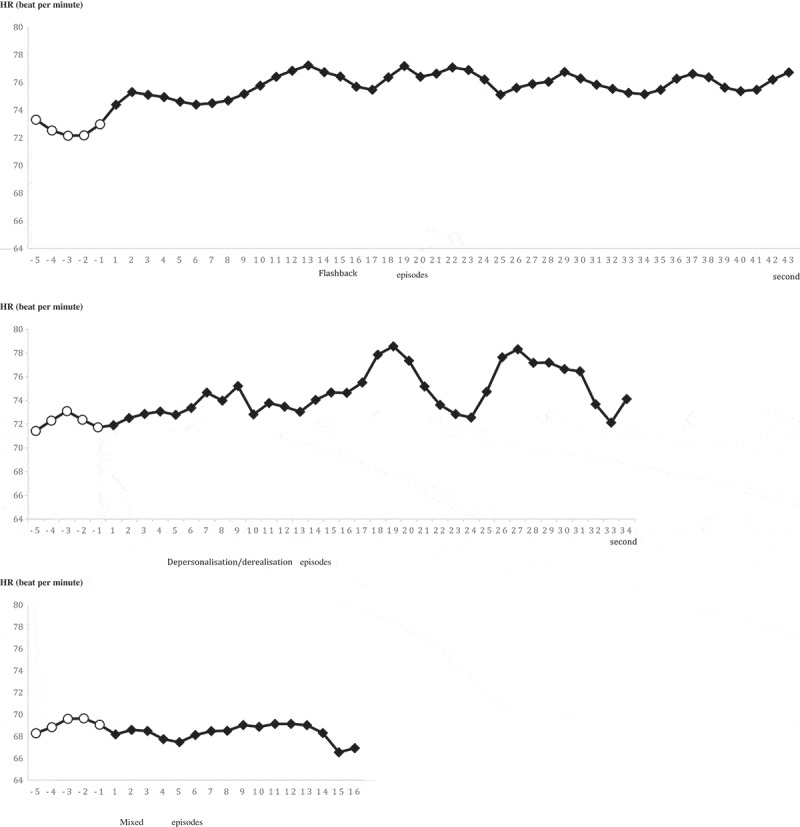
Second-by-second heart rate during flashback, depersonalisation/derealisation, and mixed episodes. Note 1: Seconds −5 to −1 shown here are the five seconds of ordinary recall heart rates just before flashbacks began.Note 2: The total number of flashback, depersonalisation/derealisation, and mixed episodes involved in this figure were 114, 20, and 19, respectively. Due to varying lengths of the episodes, different percents of the overall data points were involved in calculating the mean HR. For flashback: S1–S10: > 98%; S11–S20: > 43%; S21–S30: > 29%; S31–S43: > 20%. For depersonalisation/derealisation: S1–S10: > 50%; S11–S20: > 40%; S21–S34: > 20%. For mixed periods: S1–S10: > 53%; S11–S16: > 20%.

Figure 3 shows the fluctuation of HR during the first 43 seconds of flashbacks, 34 seconds of depersonalisation/derealisation, and 16 seconds of mixed episodes. As noted in this figure, the mean HR during later time points were estimated by a decreasing percentage of the overall data points. These lengths were chosen to present estimations based on no less than 20% of the data points.

## 4. Discussion

Heightened activation of the SNS has been commonly found as a reaction to involuntarily encountering reminders of traumatic events among individuals with PTSD (e.g. Ehlers et al., 2010; Hetzel-Riggin, 2010). However, very little research has examined how the SNS and PNS respond to voluntary recall of trauma, which is of great clinical interest since it is a central procedure in exposure-based therapies. Adding to the literature, our results demonstrate for the first time changes in PNS activity (as indexed by HRV measures), and associations between HR and different psychological responses during the process of voluntarily retrieving trauma memories. We now discuss these findings on HRV and HR.

### 4.1. Changes in HRV

Over the period of voluntary trauma recall, a significant elevation of HF-HRV was found, suggesting an increase in PNS activity. This finding is inconsistent with a previous study which found a decrease in PNS activation among individuals with PTSD (Keary et al., 2009). The inconsistency may be due to differences in study design: we adopted an active control condition (i.e. neutral recall), whereas Keary and colleagues (Keary et al., 2009) compared measures during trauma recall against a resting period. Since the action of speaking and the cognitive activities involved in memory recall have significant effects on cardiovascular measures (Hauschildt, Peters, Moritz, & Jelinek, 2011), differences between the two studies may be at least partially associated with the selection of a control condition.

Significant associations were not evident between heightened HF-HRV and the psychological measures included in the study. However, the significant overall increase in state depersonalisation/derealisation linked to trauma recall was correlated with a decrease in LF/HF-ratio, suggesting PNS dominance over the SNS. The result supports previous findings of an association between increased PNS activity and passive defense mechanisms (Richter, Schumann, & Zwiener, 1990). Future studies with bigger sample sizes and subgroups varying in depersonalisation/derealisation tendencies are warranted to further examine the relationship between HRV measures and psychological phenomena during voluntary trauma recall.

### 4.2. Changes in HR

Contrary to our hypothesis, average HR during trauma recall was not significantly higher than during neutral recall. This may be due to the lengthier period of trauma recall and, perhaps more importantly, to the heightened calming effect of the PNS, which lowered HR as recall progressed (see Figure 2).

Multiple associations between changes in HR and in psychological states were found. First, during trauma recall, greater HR decreases (or milder HR increases) relative to neutral recall were significantly correlated with greater increases in fear and perceived threat. Decrease in HR has been associated with both inhibited defensive responses, such as freezing, to highly stressful situations (Graham & Clifton, 1966 ; Holmes, Brewin, & Hennessy, 2004), or an attentive mental state of higher cognitive load (Sokolov, 1960). Our finding is more likely to reflect a passive defensive response (e.g, freezing). Although contrary to our original hypotheses, these findings are reminiscent of Holmes and colleagues’ (Holmes et al., 2004) study, in which HR decreases were associated with freezing behaviours during moments of stress that later came back as involuntary memories.

However, similar to a previous study (Halligan et al., 2006), we did not find a significant correlation between variations in HR and in state depersonalisation/derealisation, an extreme form of passive defensive response. It is possible that while lowered HR may be indicative of a passive fear response, it may not necessarily suggest an altered state of consciousness, such as depersonalisation or derealisation. Instead, our data showed a significant periodic elevation in HR during the brief moments when flashbacks first occurred, and this elevation appeared to be maintained if flashbacks continued.

Overall, our findings suggest the potential of utilising HR to confirm important psychological reactions in exposure therapy. Our findings differ from Halligan and colleagues’ (Halligan et al., 2006) study, in which no significant correlations between HR and subjective feelings were found. The inconsistency may be related to differences in study design. For example, we only included individuals with PTSD, whereas Halligan and colleagues (Halligan et al., 2006) included trauma-exposed individuals without PTSD as well. We adopted an active control condition and assessed specific psychological states (e.g. fear and perceived threat), whereas Halligan and colleagues (Halligan et al., 2006) adopted a resting baseline as the control condition and asked participants to give a general rating of distress. These different aspects of the current study may have increased the sensitivity of the measures.

### 4.3. Limitations and future research directions

The first methodological limitation of the current study is its small sample size, which has restricted the statistical power to conduct more complete and sophisticated analyses. For example, some participants experienced depersonalisation/derealisation and/or a mixture of depersonalisation/derealisation and flashbacks. However, the number of these participants was too small to analyse. Second, we asked participants to identify exactly when each of their flashback periods started. Imprecision in their reports would have caused noise in our data. For this reason, and the fact that this is one of the first studies examining real-time HR associated with flashbacks, future replication is warranted.

Third, we targeted the first five seconds of each flashback period since more data points were available within this window. While our study identified an HR elevation during this initial period, this finding is mainly associated with the response of the PNS because the SNS response may take longer (i.e. up to five seconds) to occur (Hainsworth, 1995). It would be valuable to examine how HR continues to change beyond the initial five seconds of flashbacks. Similarly, it would be of clinical relevance to understand how flashbacks of different length vary in terms of their psychological nature (e.g. content and emotional valence). These more sophisticated analyses require larger-scale investigations in the future.

Fourth, we used an active control condition (i.e. neutral recall) as opposed to a pure resting baseline. This design accounted for the potential influence of movement (i.e. the act of speaking) on the cardiovascular measures. Future studies should further evaluate and account for other factors related to movement, such as the speed of speech and body movement. Fifth, most of our participants were on medication, some of which may have affected cardiovascular activity. Although effects of medication have been addressed by our within-subject design, this issue should be examined more systematically in future studies.

Finally, we adopted a cross-sectional study design, which did not allow direct examination of the long-term implications of our findings. For example, despite the finding of an association between heightened PNS dominance and greater state depersonalisation/derealisation during trauma recall, the current study could not determine whether these phenomena predicted poorer responses to exposure therapy. Follow-up studies with longitudinal designs are required to explore the practical clinical applications of cardiovascular indices.

Overall, the current study adopted a novel design to assess relationships between psychological and cardiovascular responses to the voluntary recall of trauma. The results suggest cardiovascular measures have potential in clinical settings as a way to detect clinically significant psychological states such as flashbacks. The link between increased PNS dominance and dissociative reactions emphasises the need to pace exposure carefully so that it does not inadvertently lead to freezing and shut-down. As well as leading to better understanding of the complex psychological processes involved in exposure-based therapies, the results may inform future advances such as the development of biofeedback techniques to achieve optimal levels of reactivity during exposure.
